# The vertebrate- and testis- specific transmembrane protein C11ORF94 plays a critical role in sperm-oocyte membrane binding

**DOI:** 10.1186/s43556-022-00092-1

**Published:** 2022-09-02

**Authors:** Hongying Hao, Baolu Shi, Jiacheng Zhang, Ao Dai, Wenhao Li, Haidi Chen, Wenya Ji, Chenjia Gong, Chang Zhang, Jing Li, Li Chen, Bin Yao, Peng Hu, Hao Yang, Juergen Brosius, Shanshan Lai, Qinghua Shi, Cheng Deng

**Affiliations:** 1 Institutes for Systems Genetics, Frontiers Science Centre for Disease-Related Molecular Network, National Clinical Research Centre for Geriatrics, West China Hospital, Sichuan University, Chengdu, 610212 Sichuan China; 2 Jiangsu Key Laboratory for Biodiversity and Biotechnology, College of Life Sciences, Nanjing Normal University, Nanjing, 210023 China; 3 Division of Reproduction and Genetics, First Affiliated Hospital of USTC, School of Basic Medical Sciences, Division of Life Sciences and Medicine, Hefei National Research Centre for Physical Sciences at the Microscale, Biomedical Sciences and Health Laboratory of Anhui Province, University of Science and Technology of China, Hefei, China; 4 Key Laboratory of Exploration and Utilization of Aquatic Genetic Resources (Ministry of Education), Shanghai Ocean University, Shanghai, China; 5 State Key Laboratory of Reproductive Medicine, Nanjing Medical University, Nanjing, 210029 China; 6 Center of Reproductive Medicine, Nanjing Jinling Hospital, Clinical School of Medical College, Nanjing University, Nanjing, 210002 Jiangsu China

**Keywords:** *C11orf94*, Male fertility, Fertilization, Sperm-oocyte binding, Sperm-oocyte fusion, Horizontal gene transfer

## Abstract

Sperm-oocyte membrane fusion is necessary for mammalian fertilization. The factors that determine the fusion of sperm with oocytes are largely unknown. So far, spermatozoon factor IZUMO1 and the IZUMO1 counter-receptor JUNO on the oocyte membrane has been identified as a protein requiring fusion. Some sperm membrane proteins such as FIMP, SPACA6 and TEME95, have been proved not to directly regulate fusion, but their knockout will affect the fusion process of sperm and oocytes. Here, we identified a novel gene *C11orf94* encoding a testicular-specific small transmembrane protein that emerges in vertebrates likely acquired via horizontal gene transfer from bacteria and plays an indispensable role in sperm-oocyte binding. We demonstrated that the deletion of *C11orf94* dramatically decreased male fertility in mice. Sperm from *C11orf94*-deficient mice could pass through the zona pellucida, but failed to bind to the oocyte membrane, thus accumulating in the perivitelline space. In consistence, when the sperm of *C11orf94*-deficient mice were microinjected into the oocyte cytoplasm, fertilized oocytes were obtained and developed normally to blastocysts. Proteomics analysis revealed that *C11orf94* influenced the expression of multiple gene products known to be indispensable for sperm-oocyte binding and fusion, including IZUMO1, EQTN and CRISP1. Thus, our study indicated that C11ORF94 is a vertebrate- and testis-specific small transmembrane protein that plays a critical role in sperm binding to the oolemma.

**Supplementary Information:**

The online version contains supplementary material available at 10.1186/s43556-022-00092-1.

## Introduction

Fertilization is a vital biological process, fusing male and female gametes to form a zygote. Spermatogenesis occurs in the seminiferous tubules of the testis and the process can be divided into three stages: mitotic cell division, meiosis and spermiogenesis [1, 2]. Dramatic morphological changes will occur during spermiogenesis, transforming round spermatids into elongated, tadpole-like spermatozoa with only one-fifth of their original sizes [3]. Thousands of germ cell-specific genes are highly expressed in haploid germ cells and participate in spermiogenesis [4, 5]. In the past 15 years, gene knockout studies have identified 20 male germ cell specific genes or gene subtypes that play a crucial role in spermatogenesis [6]. In mammals, the oocyte is characterized by rich microvilli on the membrane surface [7]. Sperm-oocyte binding and fusion mainly occurs in this area, emphasizing its importance for fertilization [4].

Fertilization is necessary for mammalian reproduction and this process includes many important events, such as sperm capacitation, zona pellucida (ZP) binding, acrosome reaction, ZP penetration, sperm-oocyte binding, gamete membrane fusion, and pronucleus formation [8, 9]. In order to acquire the ability for fertilization, mature spermatozoa undergo a series of biochemical transformations in the female reproductive tract, which is called capacitation [10, 11]. Capacitated spermatozoa exhibit a vigorous swimming pattern described as hyperactivation [12]. This condition allows spermatozoa to penetrate the uterine and tubal viscoelastic fluid more efficiently [13]. If the sperm cannot swim freely, it will attach to the oviductal wall, so it cannot progress along the fallopian tube and interact with the oocyte, resulting in fertilization failure [14]. The capacitated sperm release hyaluronidase to penetrate the extracellular matrix of the cumulus cells that surrounds the ovulated oocyte. After receptor binding to the ZP3 glycoprotein, binding to ZP3/ZP4 induces the acrosome reaction. [9]. A variety of enzymes, such as proteases, glycosidases, phosphatases etc. Contained in the acrosomal vesicle are released, dissolve locally the ZP, and form an entry channel towards the oocyte [15]. Another important function of the acrosome reaction is to induce changes in the sperm membrane, especially the locations of proteins crucial for sperm-oocyte binding and fusion, such as the izumo sperm oocyte fusion 1 (IZUMO1) and sperm acrosome associated 6 (SPACA6) [16, 17]. After the acrosome reaction, the sperm reaches the perivitelline space (PVS). At this time, the sperm plasma membrane located in the postacrosomal segment of the sperm head fuses with the oocyte plasma membrane (oolemma) where microvilli are in abundance [18], and then extends to the area behind the head. At the same time, when the sperm contacts the microvilli-rich region it will trigger the zona reaction [19–21] and prevent other sperm from entering the cell [22], in order to avoid additional sperm binding to the ZP, thus preventing the production of polyploid embryos [23, 24]. Sperm lacking either membrane proteins IZUMO1and SPACA6, transmembrane protein 95 (TEME95) and sperm-oocyte fusion required 1 (SOF1) is able to penetrate the ZP but fail to fuse with the oocyte, leading to sperm accumulation in the PVS and consequently to fertilization failure [25–27]. Clinically, the inability of sperm to recognize the oocyte plasma membrane will lead to male infertility. For this disorder, intra-cytoplasmic sperm injection (ICSI) is used to achieve in vitro fertilization (IVF) [28].

The C*11orf94* gene is located on chromosome 11 in humans and mainly expressed in testicular tissue (data from NCBI, fertility online), but its function has not been established, as of yet. The homologous gene in mice is *1700029I15Rik*, and for simplicity, here we will use a designation for the mouse homologue that is similar to the human gene, namely *C11orf94*. Here, we demonstrated that C11ORF94 is a small transmembrane protein that mainly exists on the membrane of round spermatids, and its expression fades after sperm maturation. In order to study the physiological function of C11ORF94, we constructed a *C11orf94* gene knockout mouse line and conducted a comprehensive study on sperm number, morphology and fertilization. Our results might be of significance for the diagnosis and treatment of male infertility.

## Results

### C11ORF94 *is a* vertebrate- and testis-specific small transmembrane protein

**Fig. 1:**
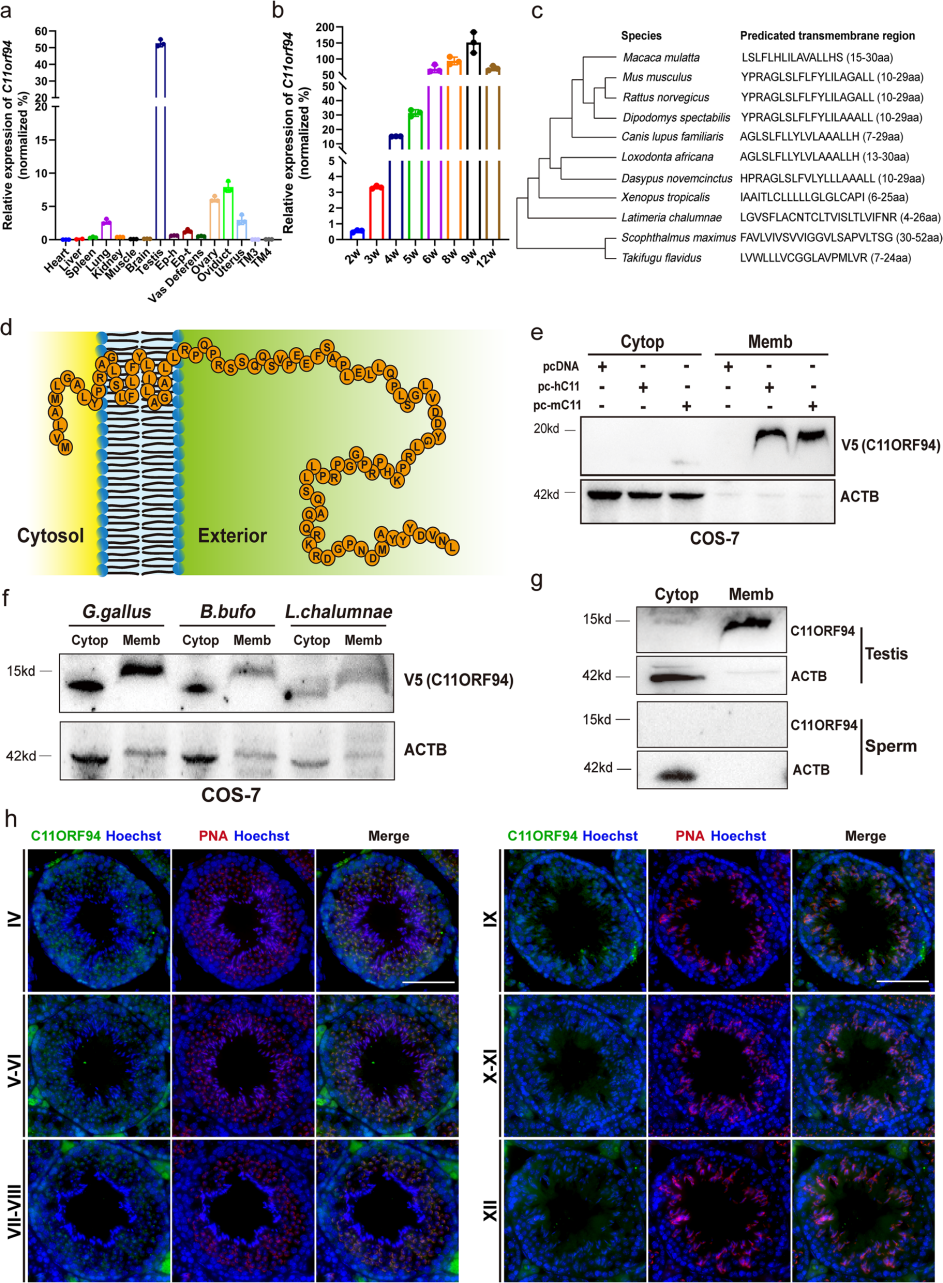
C11ORF94 is a vertebrate derived transmembrane protein highly expressed in testis.  **a** Analyses of mRNA expression pattern of *C11orf94* in mice by Real-time PCR. Mouse *C11orf94* gene is highly expressed in testis (*n* = 3). TM3, Leydig cell line; TM4, Sertoli cell line. **b** Detection of *C11orf94* mRNA expression in testis at different times after birth (*n* = 3). Mouse *C11orf94* gene expression was first detected 3 weeks after birth. **c**-**d** Prediction of transmembrane region of C11ORF94 in vertebrates. The colour map shows the transmembrane distribution of mouse C11ORF94 protein. The N-terminal is located inside the cell and the C-terminal is located outside the cell. **e**–**g** Western blot detection of C11ORF94 expression in cell membrane components in testis, sperm and COS-7 cells. β-Actin was used as loading control. Endogenous C11ORF94 is about 15 kDa, while transfected C11ORF94 (HA) is about 20 kDa, β-Actin present in all samples (~ 42 kDa). **h** Immunofluorescence was used to detect the C11ORF94 expression in wildtype testis from spermatogenesis stage IV to stage XII. C11ORF94 appears green, PNA appears red. Nuclei were stained with Hoechst33342. Scale bar, 100 μm

**Fig. 2:**
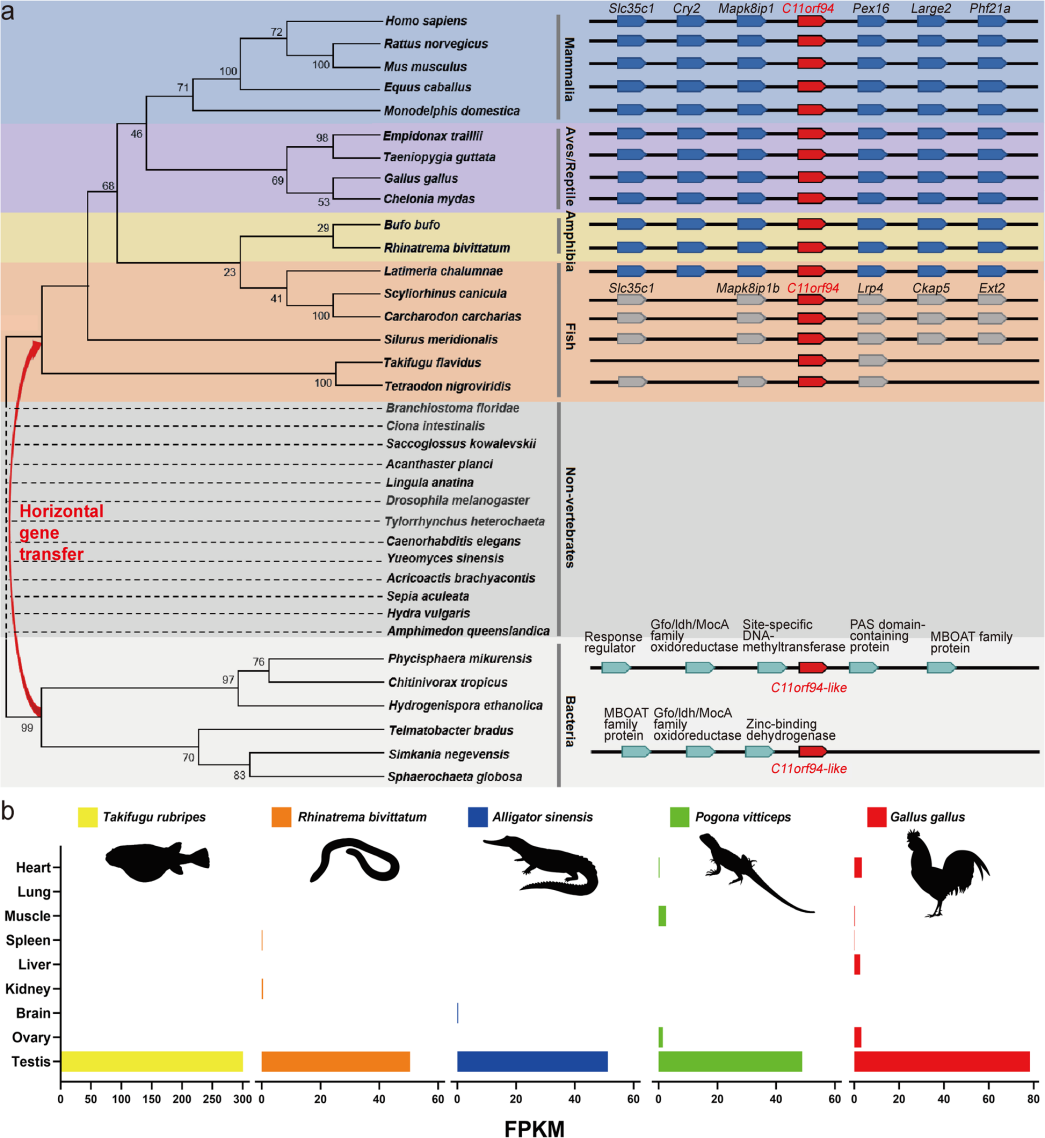
Conservation analysis of *C11orf94* during evolution. **A** Phylogenetic analysis showed that the *C11orf94* gene exist in vertebrates and maybe horizontally transferred from a bacterial genome. Synteny for chromosomal regions containing the *C11orf94* gene. The *C11orf94* gene and the neighbouring genes in vertebrate genome fragments are shown on right. **b** RNA-sequencing data acquiring from an existing database indicated that *C11orf94* is highly expressed in testicular tissues in vertebrates. Animal profiles were downloaded from PHYLOPIC (http://phylopic.org/image/browse)

The human *C11orf94* gene harbours an open reading frame of 98 amino acids and is located on chromosome 11. Our database (https://mcg.ustc.edu.cn/bsc/spermgenes2.0/index.html) shows that it is predominantly expressed in human testis. The murine ortholog for human *C11orf94* is *1700029I15Rik*, which is located on chromosome 2 also being predominantly expressed in the in testes [29]. Real-time quantitative PCR results further indicated that C*11orf94* is highly expressed in testes of male mice and weakly expressed in the female reproductive system, but not expressed in either mouse Sertoli cell line (TM4 cell line) or Leydig cell line (TM3 cell line) (Fig. 1a). *C11orf94* was initially detected in testes of 3-week-old mice, at which time round spermatids are in active development, and its expression increased gradually as time went on, suggesting that C11ORF94 is involved in spermiogenesis (Fig. 1b). Because there is little research on this protein, we queried the DTU website (https://services.healthtech.dtu.dk/service.php?TMHMM-2.0), which predicts that C11ORF94 is a transmembrane protein with an N-terminal transmembrane domain in multiple species (Fig. 1c and d). To ascertain the membrane locating pattern of C11ORF94, we separated the membrane and cytosol fraction from COS-7 cells after overexpressing C*11orf94* of human, mouse and several other vertebrates, and found that C11ORF94 was mainly detected in the cell membrane (Fig. 1e and f). Furthermore, we separated the membrane and cytosol fraction of mouse testis cells and spermatozoa from caudal epididymis through ultracentrifugation and found that the vast majority of endogenous C11ORF94 is located on the membrane of testis cells rather than in cytoplasm. Interestingly, C11ORF94 is undetectable in mature sperm cytoplasm or membranes (Fig. 1g). In order to verify the location of C11ORF94 in testes, we performed Immunofluorescence (IF) using an antibody raised against a synthetic 15 amino acid peptide from the section aa32-aa46 of mouse C11ORF94 and found that C11ORF94 signal mainly appears in seminiferous tubules during spermatogenesis stage IV-VIII and the signal vanishes beginning at stage IX (Fig. 1h). These phenomena indicate C11ORF94 locates at the developing acrosome on primary round spermatids via colocalization analysis with lectin PNA, as a unique molecular probe for acrosome [30]. All above are consistent with the results of mouse single-cell RNA sequencing (Supplementary Fig. 4) [31]. Phylogenetic analysis showed that the C*11orf94* gene does not exist in non-vertebrates, but began to appear in cartilaginous fish (Fig. 2a). Multiple sequence alignments showed that the C11ORF94 protein to be conserved in all vertebrates and conserved to a higher degree in mammals (Supplementary Fig. 1a and b). Surprisingly, we found a gene with some similarities to *C11orf94* in bacteria, suggesting that the emergence of *C11orf94* in vertebrates occurred by horizontal transfer from a bacterial genome (Fig. 2a and Supplementary Fig. 1c). In order to verify the conservation of *C11orf94* in vertebrates, we also analysed the expression of *C11orf94* in torafugu (*Takifugu rubripes*), two-lined caecilian (*Rhinatrema bivittatum*), chicken (*Gallus gallus*), Chinese alligator (*Alligator sinenis*) and central bearded dragon (*Pogona vitticeps*) based on published RNAseq data (Supplementary Table 1). It was found that *C11orf94* was specifically expressed in testes (Fig. 2b). Notably, all vertebrate C11ORF94 homologues are transmembrane proteins specifically expressed in testes (Figs. 1c and 2b) and it indicated that C11ORF94 evolved with a new N-terminal transmembrane domain in vertebrates. Hence, C11ORF94 originated from a bacteria hypothetical protein, is a small protein with an N-terminal transmembrane domain and specifically expressed in the membrane of round and elongated spermatids in mice.

### No abnormalities were observed in *C11orf94* knockout mice except for decreased sperm count

**Fig. 3:**
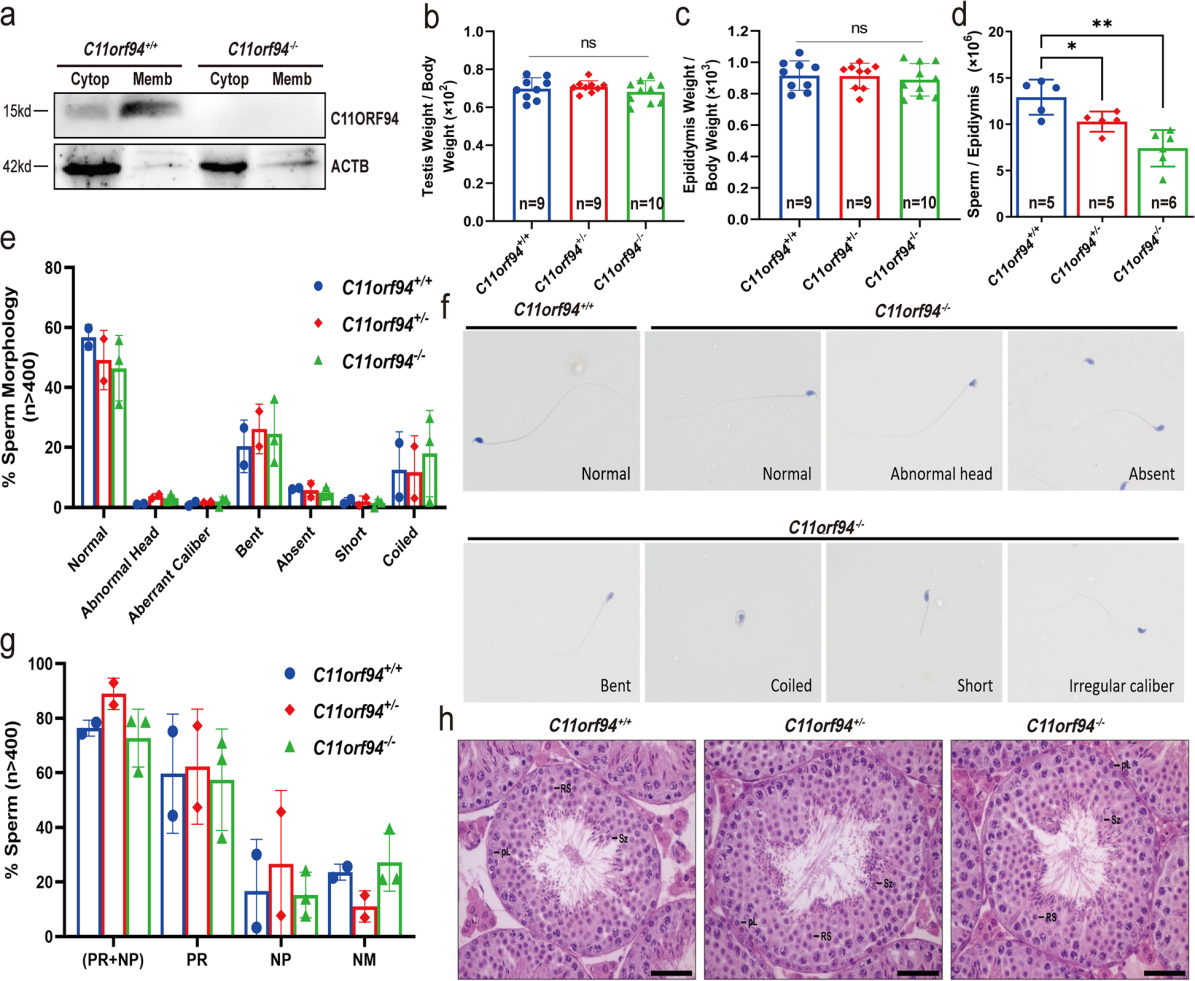
Analysis of male reproduction in *C11orf94*^*−/−*^ male mice. **a** Western blot analysis of C11ORF94 protein levels in testis of *C11orf94*^*−/−*^ and wild type mice. **b**-**c** Testis or epididymis weight/body weight. There was no significant difference between *C11orf94*^+*/*+^ and *C11orf94*^*−/−*^ male mice. [testis weight/body weight: 0.7081 ± 0.01052 (*C11orf94*^±^, *n* = 9), 0.6815 ± 0.01849 (*C11orf94*^*−/−*^, *n* = 10); epididymis weight/body weight: 0.9129 ± 0.02722 (*C11orf94*^±^, *n* = 9), 0.8891 ± 0.03275 (*C11orf94*^*−/−*^, *n* = 10)]. ns: no significance. **d** Quantitative analysis of mature sperms in the cauda epididymis of mice. This showed significant and extremely significant differences when comparing the *C11orf94*^*−/−*^ to *C11orf94*^±^ or *C11orf94*^*−/−*^ groups (*, *p* < 0.05; **, *p* < 0.01). **e**–**h** Analysis of sperm motility (PR + NP) using CASA. There was no significant difference in sperm motility among different genotypes of mice. [47.24 ± 17.56, (*C11orf94*^±^, *n* = 4); 43.13 ± 13.26, (*C11orf94*^±^, *n* = 4), sperms number > 400]. RP: Progressive motility; NP: Non-Progressive. ns: no significance. **f**-**g** Analysis of the morphology of mature sperms by HE staining. **f** is a representative diagram of various sperm morphology. **h** HE staining reveals the morphology of spermatogenesis in testis of different genotypes of mice. Pl: preleptotene; Rs: round spermatid; Sz: spermatozoa. Scale bar, 100 μm

The highly and specifically expression of *C11orf94* in testes implies that it could play an important role in male reproduction. Therefore, we generated *C11orf94* knockout mice using the CRISPR/Cas9 technique (Supplementary Fig. 2a and b), and confirmed the deletion of 38 bp in *C11orf94* exon 1 by Sanger sequencing (Supplementary Fig. 2c). Real-time PCR and Western blot analyses showed that the *C11orf94* was successfully knockout (Fig. 3a and Supplementary Fig. 2d-e). We then analysed the characteristics related to male reproduction, such as the ratio of testis or epididymis to body weight, the number of sperm in epididymis, sperm morphology and motility of the knockout mice, and compared with those of control males. There was no significant difference in testicular morphology and testis/body weight ratio between *C11orf94*^+*/*+^ and *C11orf94*^*−/−*^ mice (Fig. 3b-c and Supplementary Fig. 3), but the number of spermatozoa in the epididymis was decreased in *C11orf94*^*−/−*^ mice (Fig. 3d). Sperm morphology were assessed after hematoxylin–eosin staining of sperm smears and no significant difference was observed between *C11orf94*^*−/−*^ and C*11orf94*^+*/*+^ mice (Fig. 3e and f). Computer-aided sperm analysis (CASA) was applied to measure sperm motility and no significant difference was found in sperm motility between *C11orf94*^*−/−*^ and control mice (Fig. 3g and Supplementary Video 1). Testicular sections were stained with hematoxylin–eosin, and no obvious difference in seminiferous tubules was seen between groups (Fig. 3h). Thus, our results indicated that the deletion of *C11orf94* does not cause obvious defects in spermatogenesis, except that it reduces sperm number in mice.

### Sperm from *C11orf94*-null mice are unable to fertilize the oocyte in vivo

**Fig. 4:**
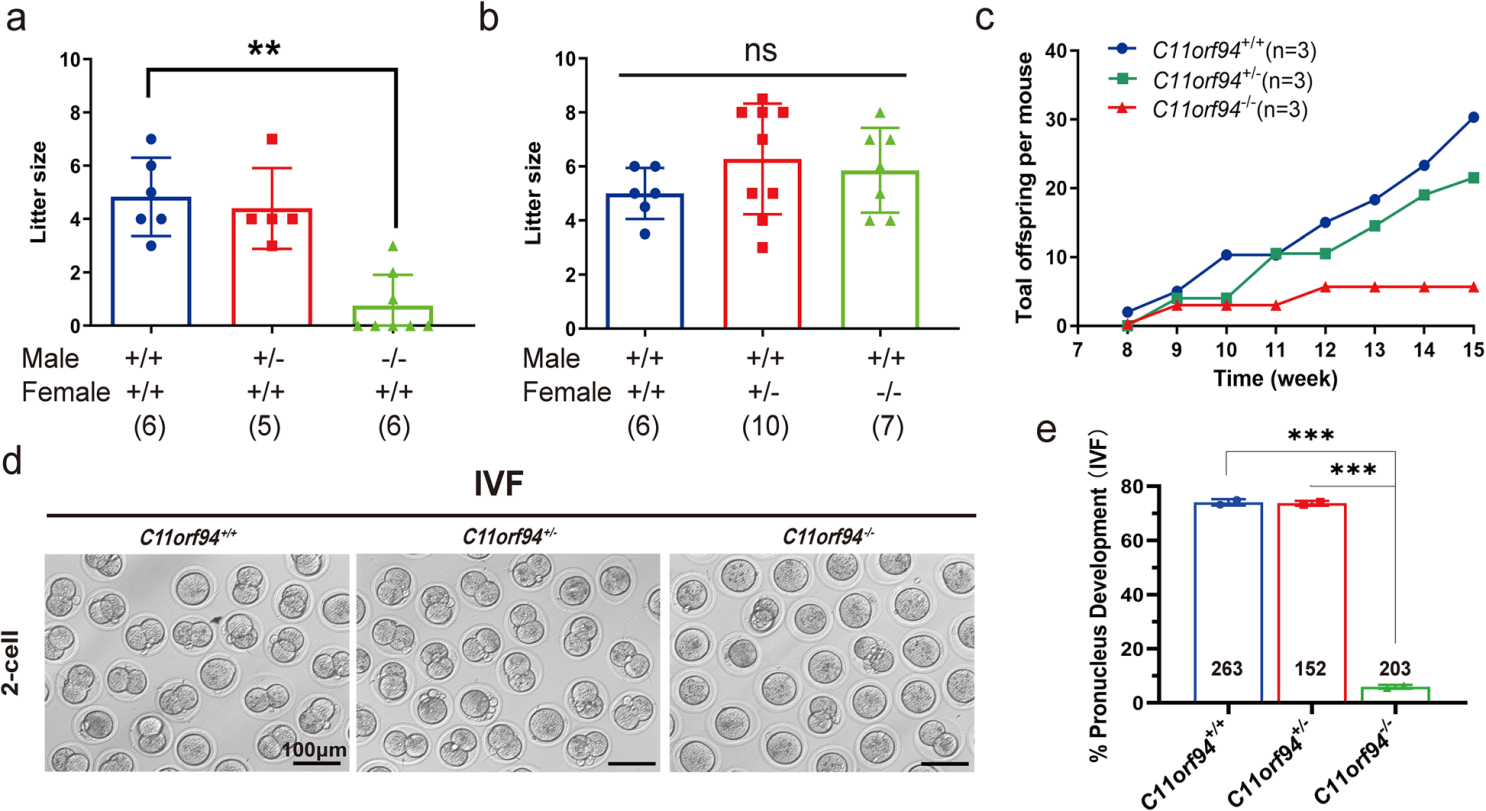
In vitro and in vivo sterility of *C11orf94*^*−/−*^ mice. **a** Litter size of crosses between C57BL/6 females (*n* ≥ 6) and *C11orf94*^*−/−*^, *C11orf94*^±^, *C11orf94*^+*/*+^ males (*n* ≥ 6). Values indicate mean ± SEM. The numbers in brackets represent the number of mating pairs (**, *p* < 0.01). **b** Litter size of crosses between C57BL/6 male mice (*n* ≥ 6) and *C11orf94*^*−/−*^, *C11orf94*^±^, *C11orf94*^+*/*+^ females (*n* ≥ 6). Values indicate mean ± SEM. The numbers in brackets represent the number of mating pairs. ns: no significance. **c** Total offspring per mouse over time for the three genotype male mice. **d** Sperm sterility with cumulus-intact oocytes by in vitro fertilization test. Sperm exact from the cauda epididymis of three genotype mice and cumulus-intact oocytes derive from wild type C57BL/6 female mice. Here is representative diagram of 2-cell in the fertilized oocyte development. **e** The ratio of pronucleus development to total oocytes. There was significant difference between *C11orf94*^*−/−*^ males and *C11orf94*^+*/*+^ or *C11orf94*.^±^ males (***, *p* < 0.005)

In order to verify whether the deletion of *C11orf94* affects reproduction, we conducted reciprocal crosses. *C11orf94*^*−/−*^ female mice could produce offspring while *C11orf94*^*−/−*^ male mice had serious defects in reproduction (Fig. 4a and b). To further verify the reduced fertility of *C11orf94*^−/−^ mice, we also conducted a successive mating experiment for adult *C11orf94*^*−/−*^ males and wild-type females. During the 2-month period of mating, we checked daily for vaginal plugs. In case mating occurred, we moved the females for separate feeding and subsequently recorded of the litter size [32, 33]. Although *C11orf94*^*−/−*^ mice exhibited normal mating behaviour and induced vaginal blockage, they were almost sterile. The number of the offspring born to *C11orf94*^*−/−*^ male mice over time was significantly lower than that of *C11orf94*^+*/*+^ mice (Fig. 4c). Similar results were obtained after IVF; the pronucleus formation rate from sperm of wild-type and heterozygous mice reached about 73% with normal embryonic development till the blastocyst stage, while the pronucleus formation rate for sperm of *C11orf94*^*−/−*^ males was ~ 5%, significantly lower than those of wild-type mice (Fig. 4d-e), which is consistent with the results of the mating experiments (Fig. 4c). These results indicate that inactivation of *C11orf94* severely reduced male fertility most probably due to defects in fertilization.

### Sperm of *C11orf94*-deficient mice fail to bind to the oolemma

**Fig. 5:**
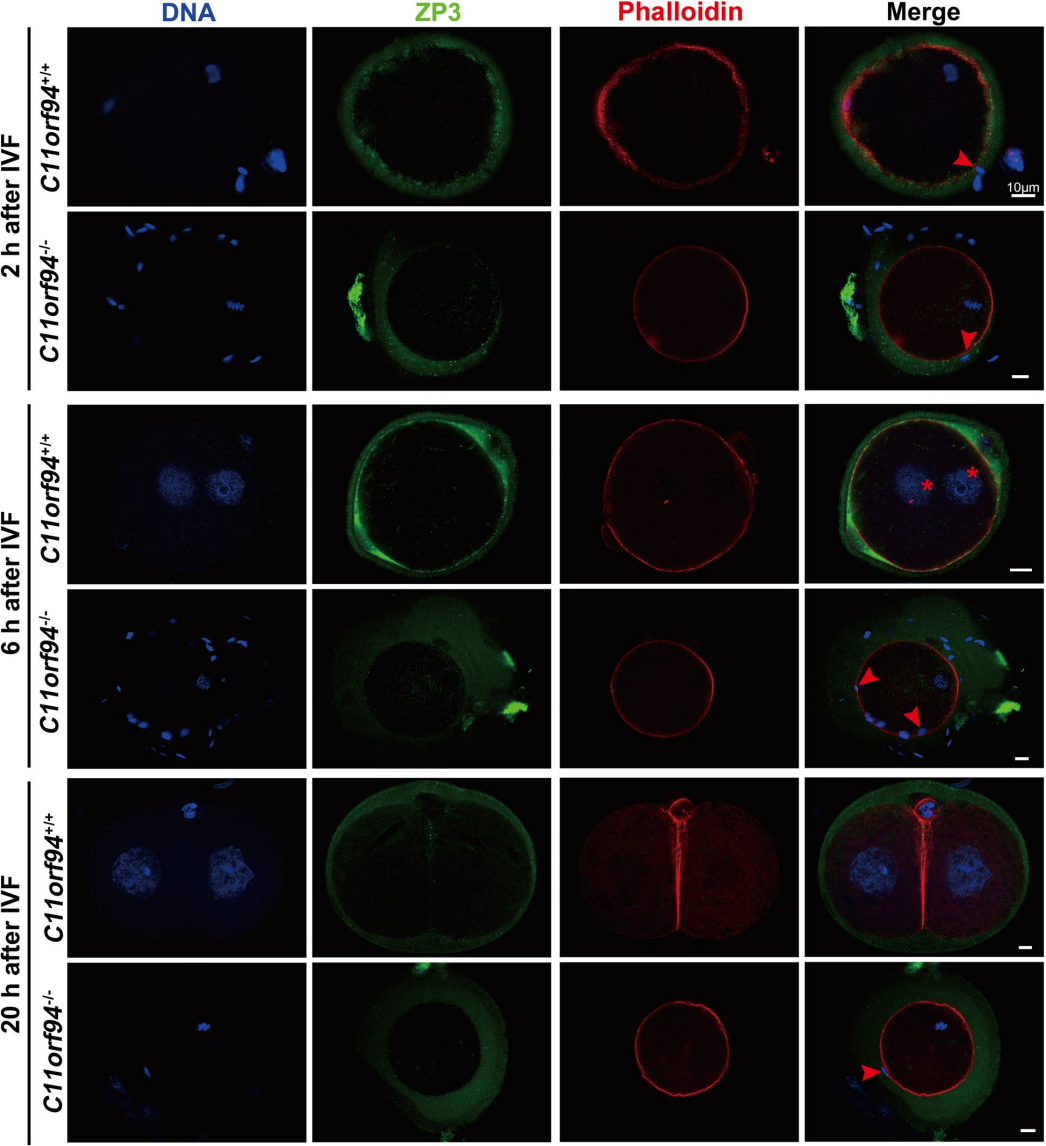
The fertilization of sperm and oocytes at different times after IVF was detected by immunofluorescence. Cumulus-intact oocytes or fertilized oocytes were stained with anti-ZP3 (green) and anti-Phalloidin (red) antibodies. DNA was counterstained with Hoechst33342 as a marker of the cell nucleus. Red arrows indicate the heads of sperms and red asterisks indicate the male and female pronucleus. Scale bar, 100 μm

**Fig. 6:**
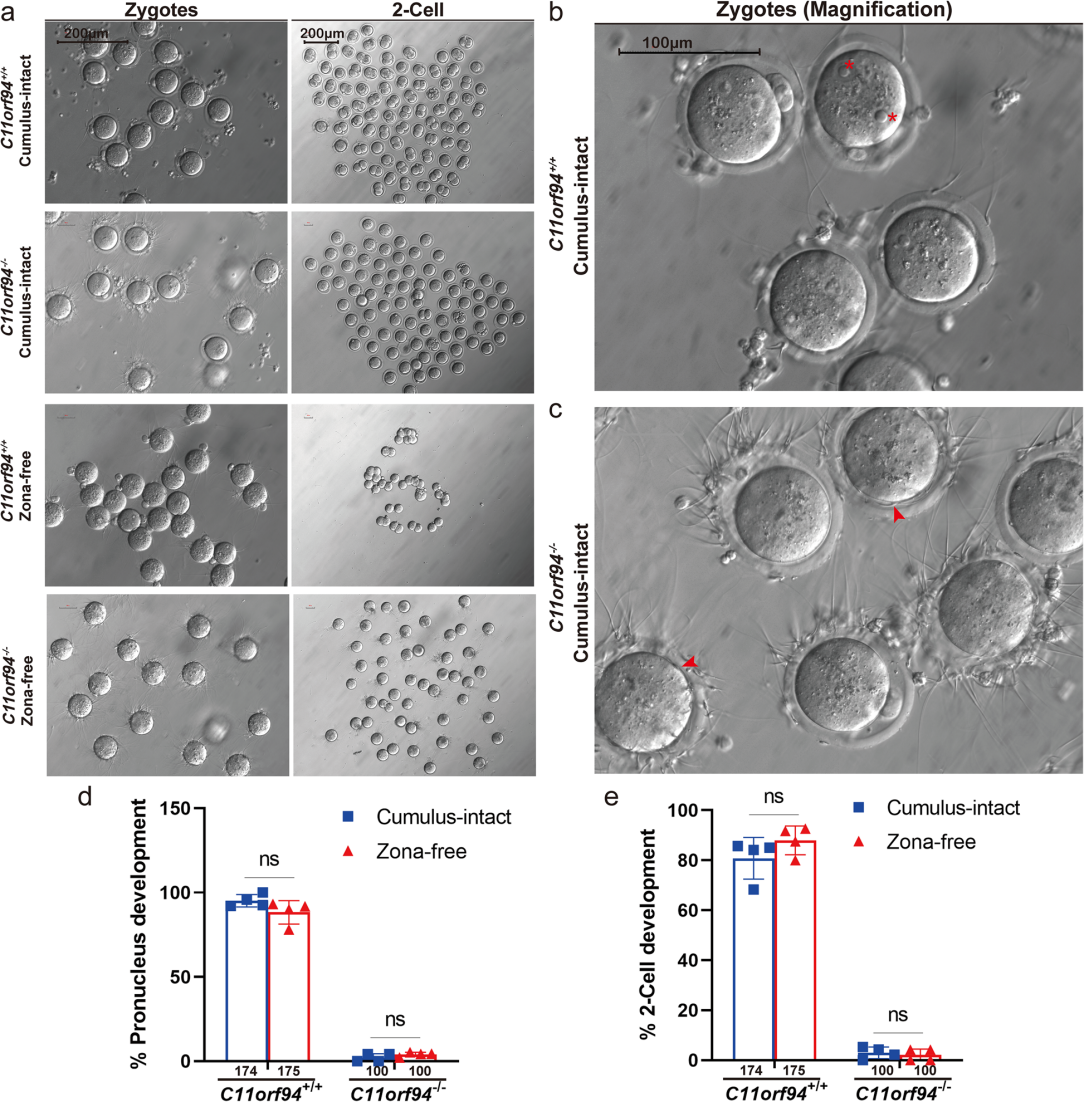
In vitro fertilization with cumulus-intact and ZP-free oocytes. **a** Microscopic image of the fertilized oocytes at zygotes or 2-cell stage. There was no observation of fertilized oocytes incubation with the sperms of *C11orf94*^*−/−*^ male mice. Scale bar, 200 μm. **b**-**c** High-resolution picture of oocytes after incubated with the sperms of *C11orf94*^+*/*+^ or *C11orf94*^*−/−*^ male mice. Asterisks indicate male and female pronucleus and arrows indicate sperms gathered in perivitelline space. Scale bar, 100 μm. **d**-**e** The ratio of zygotes or 2-cell development to total oocytes. There was no significant difference between cumulus-intact and ZP-free groups

In the process of fertilization, sperm undergoes a series of events, such as ZP binding, release of acrosomal enzymes to locally dissolve the ZP, binding and fusion with the oolemma [18, 34]. To investigate the effects of *C11orf94* deletion on fertilization, we performed IF staining for the ZP and oocyte membrane of fertilized oocytes at different time points in the process of IVF to assess the relative position of sperm and oocyte membrane and the developmental states of the fertilized oocytes. Two hours post IVF, both *C11orf94*^+*/*+^ and *C11orf94*^*−/−*^ mouse sperm passed through the ZP (Fig. 5). After 6 h, sperm from *C11orf94*^+*/*+^ mice had successfully fertilized the oocyte, and male and female pronuclei appeared, while spermatozoa of *C11orf94*^*−/−*^ mice remained near the ZP or in the PVS (Fig. 5). After 20 h, the fertilized oocyte should develop to the two-cell stage [35]. The zygote derived from wild-type sperm exhibited cell division, but the sperm from *C11orf94*^*−/−*^ mice was unable to fertilize the oocyte (Fig. 5). To understand whether the failure in fertilization results from failure in penetration of the ZP, we conducted IVF experiments using ZP-free oocytes [26, 32]. Compared with the control group, sperm from *C11orf94*^−/−^ mice still showed extremely low pronucleus formation rates, with none developing to the two-cell stage (Fig. 6a-e). The enlarged pictures and movies showed that sperm were accumulating over the oocyte surface (Fig. 5c and Supplementary Video 2). These results were in line with fertilization experiments conducted with ZP-intact oocytes (Fig. 5). Therefore, our results showed that the sperm of *C11orf94*-deficient mice can pass through the ZP akin to sperm from wild-type animals, but are unable to bind to the oolemma.

### Intra-cytoplasmic sperm injection (ICSI) rescues the ability of *C11orf94-*deficient sperm to fertilize the oocyte

**Fig. 7:**
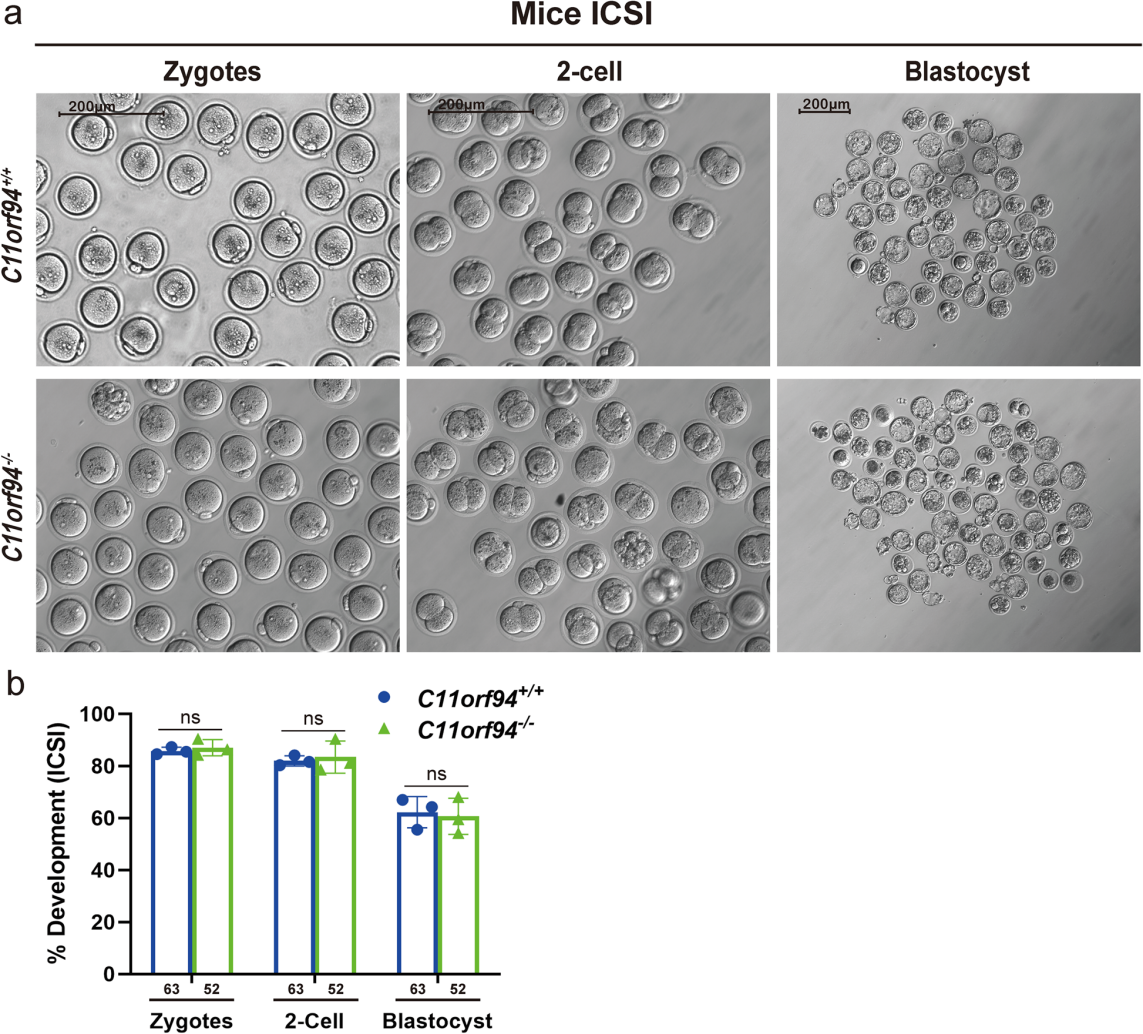
ICSI rescues the fertilizing ability of C11ORF94-deficient sperm. The *C11orf94*^+*/*+^ and *C11orf94*^*−/−*^ males mice survival rate of fertilized oocytes and representative diagram of fertilized oocytes at pronucleus stage, 2-cell and blastocyst developmental stages after ICSI. Scale bar, 200 μm

Sperm from *C11orf94*-deficient mice showed extremely low fertilization rates both in vivo and in vitro (Figs. 4 and 6). Further studies showed that the sperm from *C11orf94*^−/−^ mice could not complete the process of sperm-oocyte binding (Fig. 6c and Supplementary Video 2). Therefore, we used ICSI to let sperm from *C11orf94*^−/−^ mice bypass the process of binding and fusion with the oolemma and directly enter the oocyte cytoplasm to confirm our conclusion. As shown in Fig. 7, sperm from both wild-type and *C11orf94*^−/*−*^ mice achieved about 90% of pronucleus formation rate, and the resulted zygotes developed to blastocysts after continuation of culture. These results show that sperm of *C11orf94*-deficient mice can successfully fertilize the oocyte when they are present in the ooplasm, and confirm that they are unable to bind to the oolemma.

### *C11orf94* knockout changes the expression levels of multiple gene products involved in sperm-oocyte binding

**Fig. 8:**
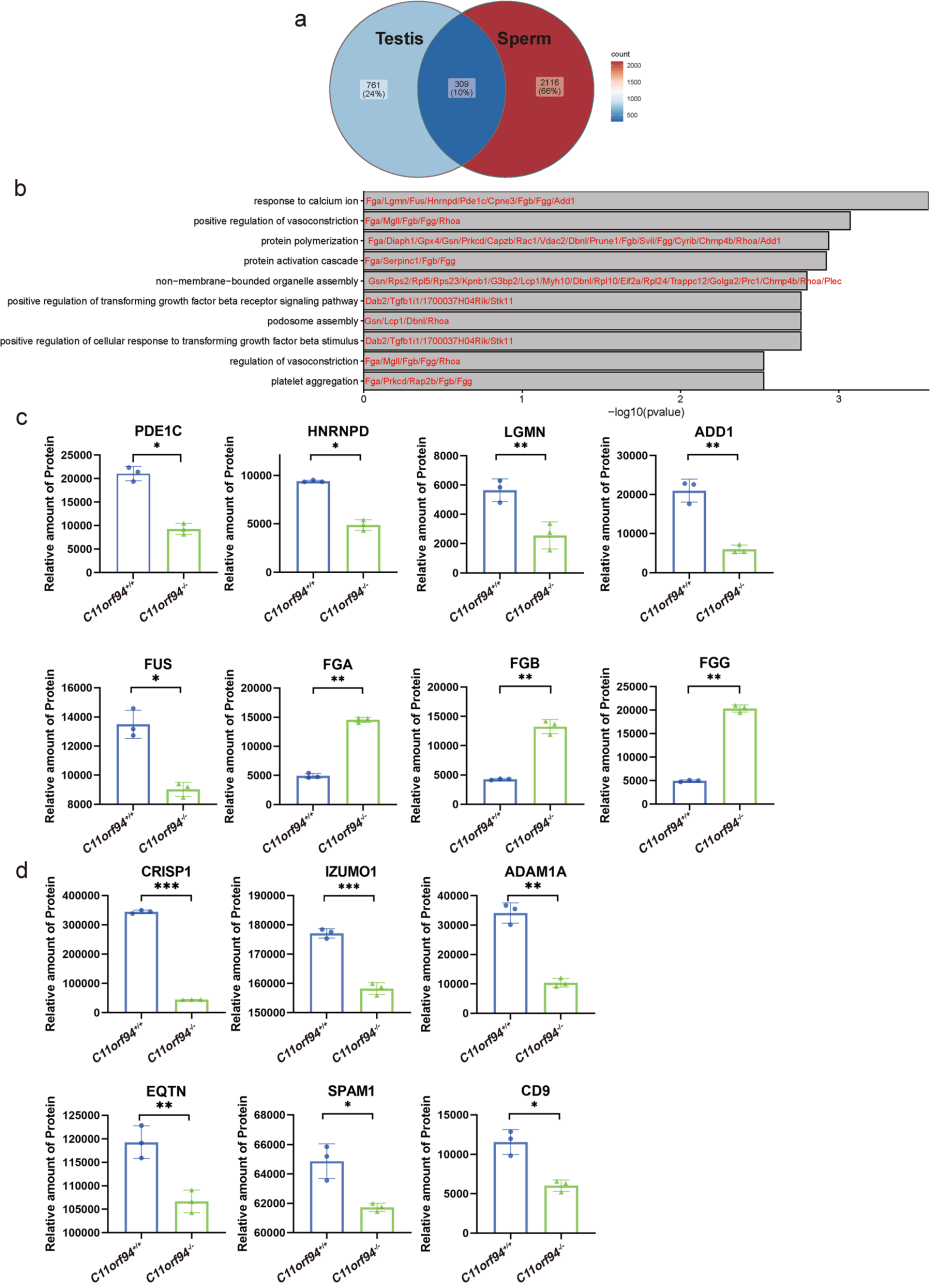
Analysis of protein changes in testis and sperm of *C11orf94*-deficient mice by mass spectrometry. **a** Venn diagram analysis of differential genes between testis and sperm. There are 1070 differential genes in testis and 2425 differential genes in sperm between *C11orf94*^+*/*+^ *and C11orf94*^*−/−*^ mice*.* **b** Go analysis of differentially expressed overlapping proteins in testis and sperm. **c**-**d** Analysis of differential genes related to Ca.^2+^ signalling pathway (**c**) and sperm-oocyte fusion (**d**) in mature spermatozoa (*n* = 3) (*, *p* < 0.05; **, *p* < 0.01; ***, *p* < 0.005)

In order to understand how *C11orf94* affects sperm-oocyte fusion, we performed mass spectrometry on testes and sperm from caudal epididymis, and then analysed the differences of protein expression in testis and sperm of *C11orf94*^+/+^ and *C11orf94*^−/−^ mice. The results showed 309 genes encoding differentially expressed proteins of testis and sperm, with most of these genes being assigned to the Ca^2+^ pathway, consistent with the fact that Ca^2+^ plays an indispensable role in spermatogenesis and fertilization (Fig. 8a-b and Supplementary Table 2). For example, ADD1, PDE1C, LGMN, HNRNPD and FUS were down-regulated in the sperm of *C11orf94*^−/−^ mice, while FGA, FGB and FGG were significantly up-regulated (Fig. 8c). Also important, we found that the expression level of some proteins involved in sperm-oocyte binding/fusion were significantly decreased in the sperm of *C11orf94*^−/−^ mice, such as IZUMO1, the cysteine rich secretory protein 1 (CRISP1), EQTN, the sperm adhesion molecule 1 (SPAM1), the disintegrin and metallopeptidase domain 1a (ADAM1a) and CD9, whereby CRISP1 was decreased most distinctly (Fig. 8d). We also analysed the expression content of these genes at different stages of spermatogenesis by using the previously published single-cell RNA-sequencing data (scRNA-seq) (Supplementary Fig. 4). Our results showed that these key genes for sperm-oocyte binding/fusion were specifically expressed in spermatids and had an expression pattern similar to *C11orf94*. Only *Crisp1* was expressed somewhat later in sperm development. For these genes related to sperm egg fusion, our RNA-seq comparison of *C11orf94*^+/+^ and *C11orf94*^−/−^ testes indicated that there are no differences at the transcript level (Supplementary Table 3). However, in *C11orf94* knockout mice the corresponding protein products were significantly reduced (Fig. 8d). Therefore, our results indicate that C11ORF94 may participate in sperm egg binding/fusion by acting as a molecular chaperone and affecting the functioning of proteins during spermatids development.

## Discussion

**Fig. 9:**
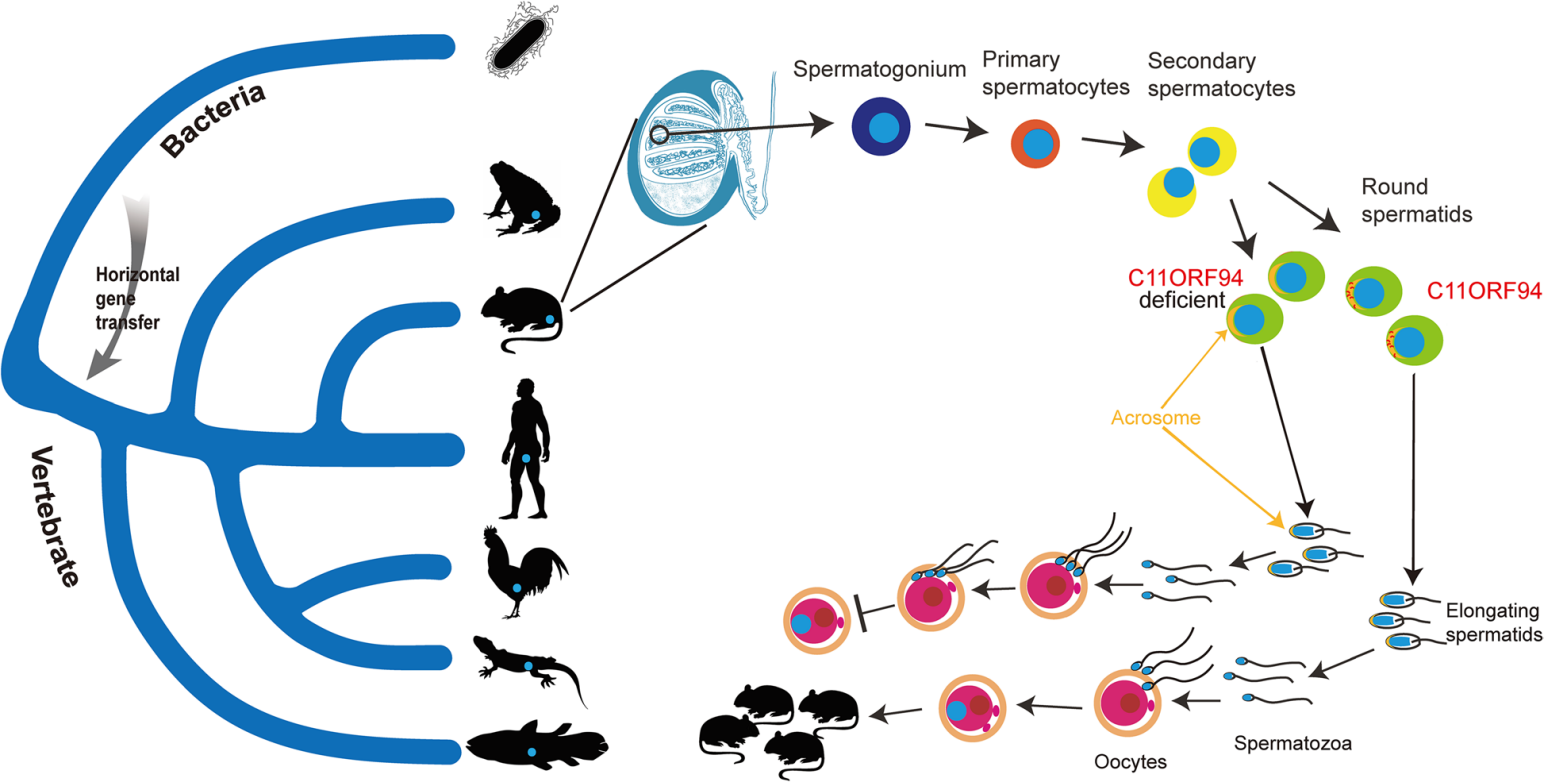
Diagram of *C11orf94* origin and function in mice. The emergence of *C11orf94* in vertebrates presumably occurred by horizontal transfer from a bacterial genome and *C11orf94* is conserved in all vertebrates. Its protein products are mainly located on the membrane surface of round spermatids in the testis and disappears from the mature sperm membrane. The absence of C11ORF94 protein allows sperm to cross the zona pellucida but it accumulates in the perivitelline space and is unable to penetrate the oolemma. Animal profiles were downloaded from PHYLOPIC (http://phylopic.org/image/browse)

C11ORF94 is a short transmembrane protein with previously unknown function. A multiple sequence alignment showed that C11ORF94 protein is conserved in the vertebrates examined and more so in mammals. It first appeared in cartilaginous fish (Fig. 2a) but surprisingly, we found a C11ORF94-like hypothetical protein in many bacteria (Supplementary Fig. 1c) and did not detect any similar sequences in all non-vertebrates examined (Fig. 2a).This phenomenon may well be due to horizontal gene transfer which, for example, recently has been shown to exert a vast influence on insect genomes [36]. *C11orf94* might be an example for horizontal transfer from bacteria, which persisted in all chordate genomes and the gene product gradually was exapted for a function in vertebrate reproduction [37]. After the horizontal gene transfer event, *C11orf94* recruited an N-terminal transmembrane domain during vertebrate evolution and is highly expressed in male testis. Deletion of *C11orf94* in mice resulted in a decrease in sperm number and severely decreased male infertility (Fig. 4). Additional studies revealed that the C11ORF94-deficient sperm remained in the PVS and eventually failed to fertilize due to its disability to bind to the oocyte (Fig. 9).

In mammals, fertilization is initially accomplished through the direct interaction between sperm and the oocyte, which is mainly mediated by gamete surface proteins. So far, the fertility test of gene knockout mice has confirmed that three genes are critical for gamete binding/fusion (CD9 and JUNO on the oocyte cell membrane, and IZUMO1 on the sperm membrane) [7, 26]. Animals lacking these proteins failed fertilization due to abnormal final adhesion/fusion steps, which had no effect on other steps of the fertilization process [38–41].

The molecular mechanism of sperm-oocyte fusion has intrigued researchers in related fields for decades [42]. The development of CRISPR technology also helped researchers to identify more gamete fusion-related factors, including the following three membrane-anchoring proteins: fertilization-influencing membrane protein (FIMP), SPACA6, TEME95 and predicted secretory proteins like SOF1, domain-containing 1 (DCST1), domain-containing 2 (DCST2) [32, 43, 44], which are essential for fertilization. Their deletions can produce phenotypes similar to IZUMO1-deficient male mice. However, an important difference between IZUMO1 and other newly discovered proteins is that heterologous cells overexpressing IZUMO1 can effectively adhere to oocytes, while the aforementioned newly discovered protein does not have this ability. These results suggest that these four proteins play little or no role in sperm-oocyte recognition. C11ORF94 is a small protein with less than 100 amino acids highly expressed in testis. The phenotype of *C11orf94* gene knockout mice is similar to the recently published knockout mice of the aforementioned four genes that can affect sperm-oocyte fusion. *C11orf94* is expressed in round and elongated spermatids, and its expression fades after sperm maturation. Through mass spectrometry, we identified 6 proteins significantly decreased in *C11orf94*-deficiency sperm that may participate in sperm-oocyte binding/fusion. *ADAM* comprises a family of transmembrane proteins with multiple conserved domains, and many members of the ADAMs are expressed in spermatogonia. Studies have shown that when some *Adam* genes (such as *Adam1a*, *Adam2* or *Adam3*) are knocked out, sperm cannot migrate from the uterus to the fallopian tube, resulting in fertilization failure [45–48]. However, our results suggest that the decrease of *Adam1a* is not the main cause of *C11orf94*-null sperm infertility. EQTN is a type I transmembrane protein mainly distributed in the acrosomal membrane. It has been reported that the deletion of EQTN coding gene *Eqtn* in mice will reduce the fertilization rate by affecting the binding between sperm and oocyte membrane [49, 50]. CRISPs family is composed of four members (CRISP1-4) in mammals. It is mainly expressed in the male reproductive tract, exists in sperm, and shows the ability of calcium channel regulation [51]. IVF experiments showed that *Crisp1* deficiency can destroy the fertilization ability of sperm using ZP-free oocytes, where the percentage of oocyte binding-fusion is significantly inhibited in a concentration dependent manner [52, 53]. Therefore, our results demonstrate that C11ORF94 is not directly involved in the interaction between sperm and oocyte membrane. The deletion of *C11orf94* leads to infertility in mice by affecting the expression of calcium pathway related proteins such as PDE1C, LGMN and ADD1, as well as sperm-oocyte fusion related proteins such as CRISP1, IZUMO1 and EQTN in mouse sperm. The specific molecular mechanism needs to be further studied.

Since sperm devoid of the *C11orf94* gene product is unable to complete sperm-oocyte fusion, we employed ICSI, an assisted reproduction technique, to overcome fertilization failure caused by non-fusion of sperm and oocytes. As expected, oocytes injected with C11ORF94-deficient sperm were successfully fertilized, normally developing to the blastocyst stage (Fig. 7), suggesting that ICSI can be used as an effective treatment for male infertility caused by abnormal expression of *C11orf94*.

In conclusion, *C11orf94,* originated in a common ancestor of vertebrates, encodes a small transmembrane protein specifically expressed in testis and plays a critical role in sperm-oocyte membrane binding., which is a key step prior to the sperm-oocyte fusion during fertilization. Our findings provide a potential basis for the diagnosis and treatment of clinical male infertility.

## Materials and methods

### Cell culture and transfection

TM3-mouse Leydig cell line, TM4-mouse Sertoli cell line and mammalian COS-7 cell lines were purchased by ATCC (American Type Culture Collection, Manassas, USA). Cells were cultured in Dulbecco’s Modified Eagle’s Medium (DMEM, Gbico) supplemented with 10% fetal bovine serum (FBS, Gbico), 100 U/mL penicillin, and 100 mg/mL streptomycin (Invitrogen). COS-7 cells were transfected with Lipofectamine 2000 (Invitrogen) according to the manufacturer’s recommendations. Plasma membrane was separated 48 h after transfection.

### Phylogenetic analyses

The *C11orf94* and *C11orf94*-like sequences were subjected to BLASTX and BLASTN (https://blast.ncbi.nlm.nih.gov) analysis. Genome Data Viewer (GDV) (https://www.ncbi.nlm.nih.gov/genome/gdv) was used to find closely related species. The sequences between the same flanking genes of closely related species were analysed to confirm once again whether there were similar sequences. C*11orf94* and *C11orf94*-like protein coding sequences were aligned by ClustalW version 1.83 with default settings, respectively. Phylogenetic trees of *C1ORF94* and C11ORF94-like proteins were constructed using neighbour-joining (NJ) algorithm with 1000 bootstrap replicates in MEGA version 7, respectively.

### Animals

C57BL/6 mice were purchased from GemPharmatech Co., Ltd., Jiangsu, China. Mice were maintained under a 12/12-h dark–light cycle at 22 °C with free access to food and water. *C11orf94*^−/−^ mice on C57BL/6 background were generated by the GemPharmatech Co., Ltd., Jiangsu, China, using the CRISPR/Cas9 technique. Several sgRNA were designed targeting the exon 1 of the mouse *1700029I15Rik* gene to generate a frameshift mutant, resulting in *1700029I15Rik* function loss. Genotyping was performed with PCRs using two primers (primer 1: 5’-GTATGGAGGACTGGTGGCAT-3’, 5’-CTGAGAGAGGCTGCAACAGTTC-3’ and primer 2: 5'-GGGCTGGACTCAGTCTCTTCCT-3’,5’-CTTGGGTCTGAGCCCATAGT-3’).

### Fertility test

Eight-week-old wide type, heterozygous and homozygous *C11orf94* knockout male mice were caged individually. Two seven-week-old female C57BL/6 mice were placed in separate male cages every week, and the vaginal plugs were checked every day to ensure successful mating. After successful mating, female mice were removed. The mating cage experiment lasted for 8 weeks, and the average litter size for each male mouse was recorded.

### RNA isolation and quantitative real-time PCR

Total RNA was extracted from mouse tissues using Trizol (Invitrogen), and cDNA was synthesized with M-MLV reverse transcriptase (Promega) and oligo (dT) mixed with random hexamer primers (Invitrogen). Real-time PCR was performed using the SYBR Green PCR kit (Vazyme Biotech Co., Ltd, Nanjing, China) and specific primers for mouse *C11orf94* (5’-CAGGGCTGGACTCAGTCTCTT-3’ and 5’-CGGGGCTGAAAATTCCTCC-3’), as well as mouse GAPDH (5’-ACCACAGTCCATGCCATCAC-3’ and 5’- TCCACCACCCTGTTGCTGTA-3’).

### Western blot

Total protein was isolated from mouse testis, sperm and transfected cells with radioimmunoprecipitation (RIPA) lysis buffer with the addition of 1 mM PMSF and 1% SDS. Samples were separated by 10% SDS-PAGE that was performed using tris–glycine or tris-tricine and transferred onto PVDF membrane. Membranes were blocked in 5% milk (diluted by PBS) for one hour at room temperature and placed overnight at 4℃ with the primary antibodies (diluted to 5% with milk). After three washes with PBST (PBS containing 0.05% Tween-20), membranes were incubated for 1 h at room temperature with secondary antibody (diluted to 5% with milk). The blots were washed three times with PBST and visualized using an enhanced chemiluminescence (ECL) Western blotting detection system (Beyotime, Shanghai, China). The antibodies used in western blots are listed in Supplementary Table 4.

### Membrane fraction preparations

Testis and sperm membrane proteins were prepared using ultracentrifugation. The testicles were homogenized by a Polytron homogenizer with lysis buffer (5% NaCl, 2% Tris–HCl, 1% EDTA in aqueous solution) on ice. For sedimentation of nuclei, the tissue homogenate was centrifuged at 400 × g for 10 min at 4℃. The resulting post-nuclear supernatant was ultracentrifuged at 100,000 g for 30 min at 4℃, the supernatant is cytoplasmic protein. The membrane pellet from the high-speed centrifugation was resuspended in lysis buffer with 1% Triton X-100, centrifuged at 25,000 g for 30 min at 4℃, the supernatant is membrane protein. Sperm and COS-7 cell membrane proteins extraction was performed using the cell membrane protein extraction kit (Beyotime Biotech, Nanjing, China), according to the manufacturer's protocols.

### Histological analysis

Adult mouse testicular tissues were fixed in Bouin’s solution overnight, then embedded into the paraffin, and sectioned at 5 μm thickness. For H&E staining, the tissue slides were deparaffinized by xylene, rehydrated with gradient ethanol, and sequentially stained with hematoxylin and eosin. After dehydration and transparency, the tissue sections were sealed with neutral resin. The images were captured via a Nikon ECLIPSE 80i microscope with a DS-Ri1 camera and processed with NIS-Elements BR software.

### Sperm quantification

Male mice were sacrificed by cervical dislocation. Epididymites were dissected and cut into small pieces into a tube containing 1 mL DMEM. Sperm were incubated at 37℃ in a 5% CO_2_ humidified incubator for 30 min to allow fully release. Sperm was counted four times using a hemocytometer under a Nikon ECLIPSE 80i microscope.

### CASA and morphological analysis

Caudal epididymis was dissected and cut, and then was placed into capacitation solution (90% Human Tubal Fluid (HTF) (Nanjing Aibei, M1150) and 10% FBS at 37℃ for 5 min for sperm release. Sperm suspension was added onto CASA sperm counting slides (MAILANG, ML CASA-60) and the movie was captured using a microscope with a high-speed camera (MAILANG, MDO6200D). Sperm motility was analysed manually. Remaining sperm suspension was centrifuged at 900 × g for 5 min, and the pellet was washed twice in PBS and smeared onto slides. After fixation in PBS-buffered 4% paraformaldehyde for 5 min, the slides were stained with hematoxylin and eosin. Sperm morphology was evaluated based on the criteria for multiple morphological abnormalities of the sperm flagella together with sperm head shape. The representative images were captured via a Nikon ECLIPSE 80i microscope with a DS-Ri1 camera and processed with NIS-Elements BR software.

### Immunofluorescence staining (IF)

*IF to testis*: Testis were fixed in 4% paraformaldehyde overnight then embedded in paraffin, and sectioned into 5 μm slices. After rehydrating the samples, retrieved antigen by boiling slices in citrate/EDTA solution (20 mg EDTA, 3 g trisodium citrate in aqueous solution) for 15 min at 75℃ and 95℃. Then 1% bovine serum albumin (BSA) in PBS was used as blocking solution for 1 h incubation. Slices were incubated with primary antibody for 1 h at 37℃ and a fluorescent second antibody/1% PNA (Invitrogen, L21409) mixture for 50 min at 37℃. Finally, samples were incubated with Hoechst33342 (Invitrogen, H1399) for 15 min subsequently washed with TBST and examined with a Nikon C2 Plus Confocal Laser Scanning Microscope system.

*IF to embryo*: Embryos were fixed in 4% paraformaldehyde for 30 min and permeabilized in PBS containing 0.5% Triton X-100 for 30 min. After incubation with 1% bovine serum albumin in PBS for 30 min, the embryos were incubated with primary antibodies for 1.5 h at room temperature (or 4℃ overnight). After three washes in PBS containing 0.5‰ Triton X-100 and 1‰ Tween-20, secondary antibodies were applied for 1 h at room temperature. Subsequently, the embryos were washed three times and mounted with the Vectashield Medium (Vector Laboratories, H-1000) containing Hoechst33342. Embryos were captured using a Nikon C2 Plus Confocal Laser Scanning Microscope system.

All antibodies used in IF are listed in Supplementary Table 4*.*

### In vitro fertilization (IVF)

Sperm was released from the caudal epididymis of male mice (2–6 months old) in HTF medium and capacitated for 30 min at 37 °C in 5% CO_2_. Ovulated oocytes from C57BL/6 female mice (3 ~ 4 weeks old) were harvested after injection of 10 IU of Pregnant Mare Serum Gonadotropin (PMSG) followed by 10 IU of human chorionic gonadotropin (hCG) 46–48 h later. The control group was placed in HTF for fertilization, while the ZP-free group was placed in Acid Tyrode's solution (Sigma) for about 1 min to remove cumulus and ZP, and then placed in HTF for fertilization. Then 4 × 10^5^/mL capacitated sperm were incubated with ovulated-oocytes in 200 μL HTF for 6–8 h at 37 °C in a humidified atmosphere of 5% CO_2_. The presence of pronuclei was scored as successful fertilization. Embryos were subsequently cultured in KSOM at 37 °C in 5% CO_2_ to obtain 2-cell and blastocyst.

### Intracytoplasmic sperm injection (ICSI)

Metaphase II oocytes, cumulus-free and zona-intact, were prepared as described above. Mouse sperms, obtained from the cauda epididymis of wild-type or *C11orf94*^−/−^ males (10 ~ 14 weeks old), were treated with ultrasound (4 × 5 s). This treatment immobilizes sperm and also results in a substantial percentage of acrosome reacted sperm whose tails are clipped off. The separation of heads and flagella was done by gentle centrifugation (300 × g for 5 min). Better survival rates are usually observed when injecting isolated sperm heads instead of a whole spermatozoon because the volume of medium entering the oocyte is much smaller. During ICSI, a single sperm head was aspirated into a thin glass microcapillary and injected into the cytoplasm of an oocyte on the stage of an inverted microscope fitted with a micromanipulator setup. The crossing of zona pellucida and plasma membrane was facilitated by an electric Piezo (Eppendorf PiezoXpert). After injection, zygotes were kept in K^+^ Simplex Optimised Medium (KSOM) (Sigma) allowing embryo development in vitro. Photographs were taken to record the development of the fertilized oocyte at various stages using a microscope.

### Statistical analysis

Results are expressed as mean ± SEM of at least three independent experiments. For statistical analysis, one-way ANOVA multiple comparison tests were performed using GraphPad Prism version 7.00 for Windows (GraphPad Software, La Jolla California USA). Differences were considered statistically significant when *p*-value < 0.05.

## Supplementary Information

**Additional file 1:** **Figure S1.** Multiple sequences alignment of C11orf94.

**Additional file 2:** **Figure S2.** Generation of C11orf94 knockout mice by CRISPR/Cas9-mediated gene targeting.

**Additional file 3:** **Figure S3.** HE staining of male reproductive tissues.

**Additional file 4:** **Figure S4.** scRNA-seq data of reproductive factors’ gene.

**Additional file 5:** **Table S1.** SRA accession number of RNA-sequencing data used in this study.

**Additional file 6:** **Table S2.** GO analysis data used in this study.

**Additional file 7:** **Table S3.** RNAseq analysis of C11orf94+/+ and C11orf94-/- testicular gene expression differences.

**Additional file 8:** **Table S4.** Antibodies used in our experiment.

**Additional file 9.**

**Additional file 10.**

## Data Availability

All data generated and analysed of this study are available from the corresponding author upon reasonable request.

## Abbreviations

ZP: Zona pellucida
PVS: Perivitelline space
aa: Amino acid
IVF: in vitro Fertilization
ICSI: Intra-cytoplasmic sperm injection
CASA: Computer-aided sperm analysis
IZUMO1: Izumo sperm egg oocyte fusion 1
JUNO: IZUMO1 receptor
FIMP: Fertilization-influencing membrane protein
SPACA6: Sperm acrosome associated 6
TEME95: Transmembrane protein 95
EQTN: Equatorin
CRISP1: Cysteine rich secretory protein 1
SOF1: Sperm-oocyte fusion required 1
DCST1: Domain-containing 1
DCST2: Domain-containing 2
SPAM1: Sperm adhesion molecule 1
ADAM1a: Disintegrin and metallopeptidase domain 1a
CD 9: CD9 protein
PDE1C: Phosphodiesterase 1C
LGMN: Legumain
ADD1: Alpha-adducin 1
