# Supplementary figures and images for: The vertebrate- and testis- specific transmembrane protein C11ORF94 plays a critical role in sperm-oocyte membrane binding
Source: Mol Biomed. 2022 Sep 2;3:27. doi: 10.1186/s43556-022-00092-1 (PMC9437168; doi:10.1186/s43556-022-00092-1)

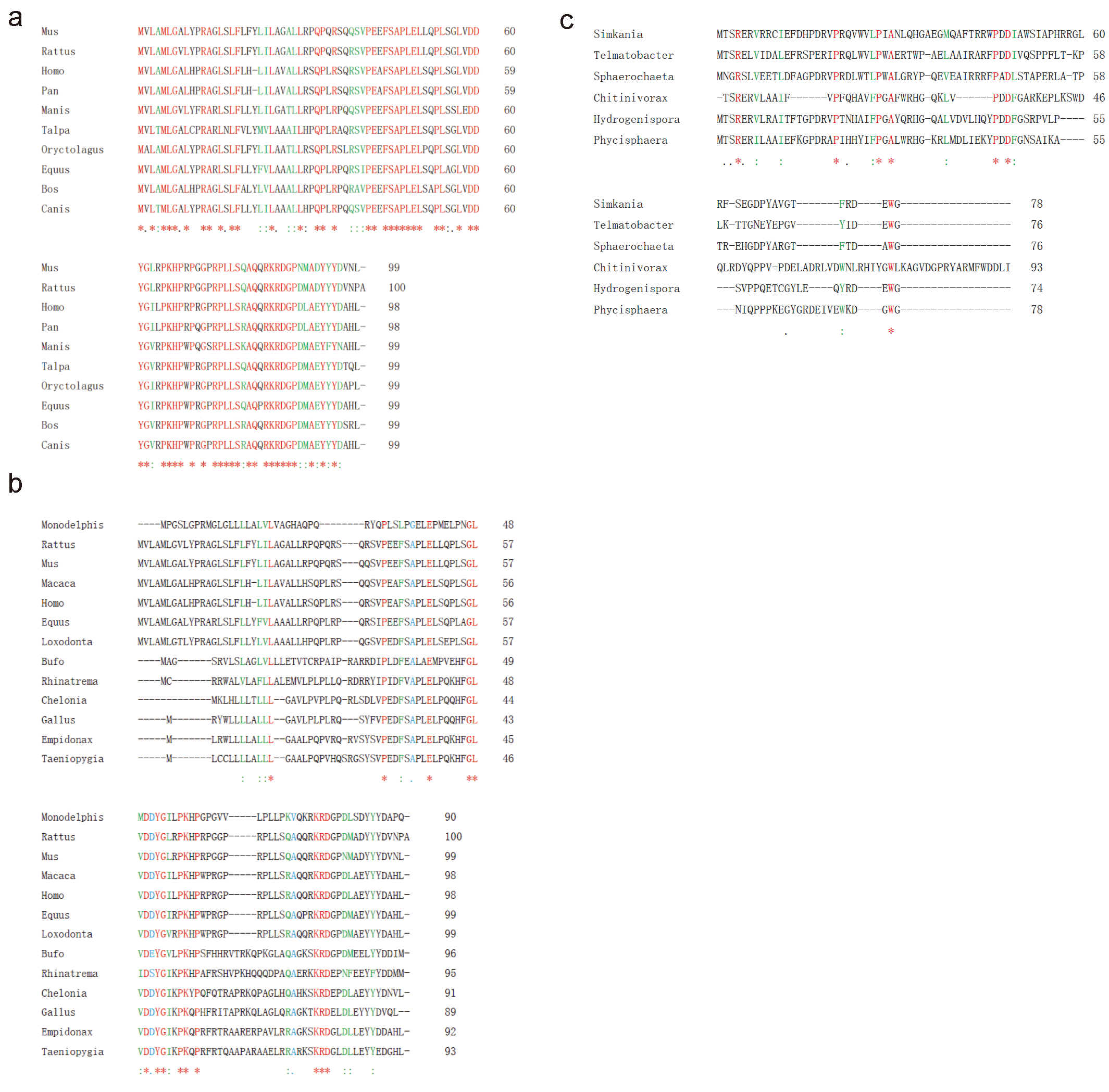

Supplement: Supplementary file 1 — Additional file 1: Figure S1. Multiple sequences alignment of C11orf94. [file 43556_2022_92_MOESM1_ESM.png]

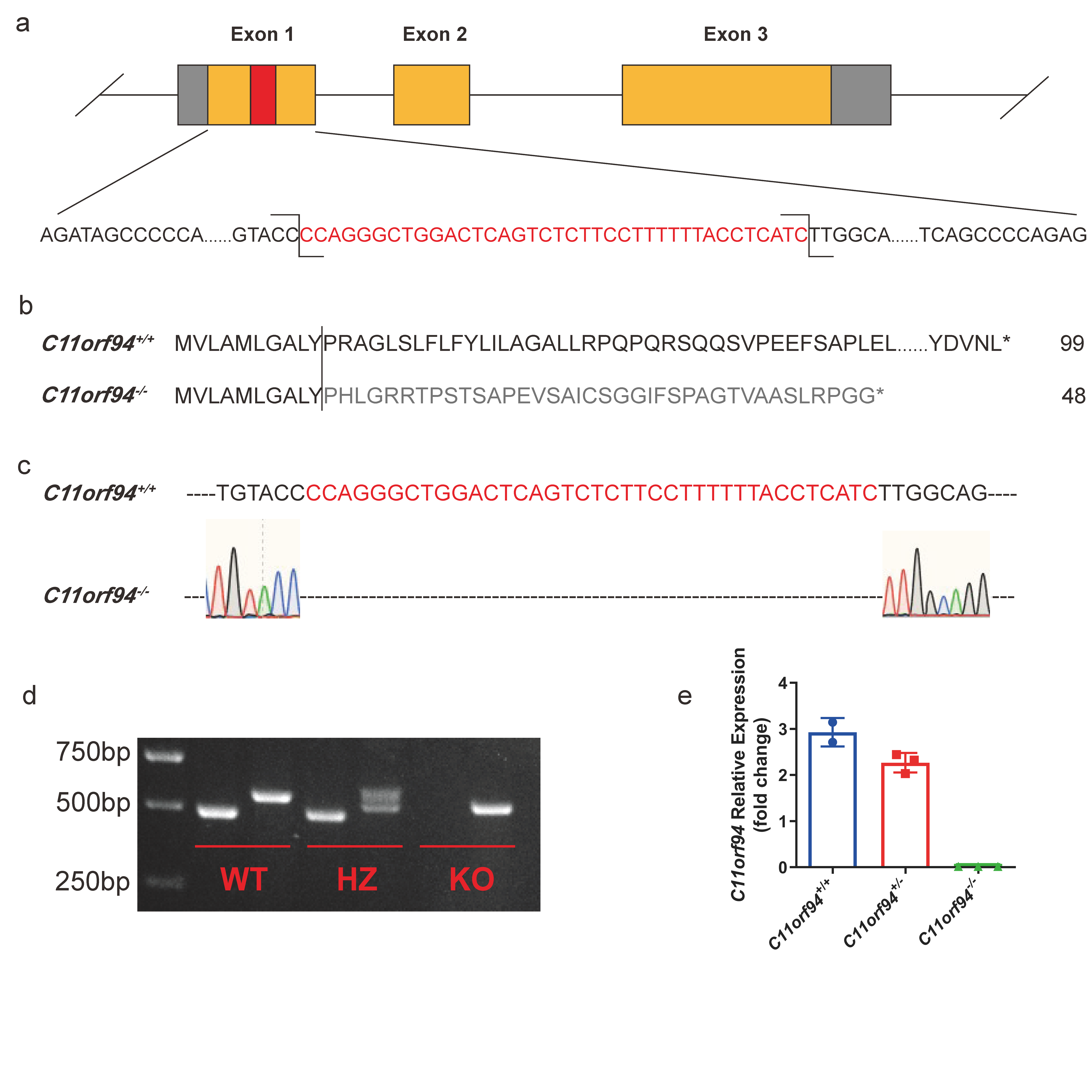

Supplement: Supplementary file 2 — Additional file 2: Figure S2. Generation of C11orf94 knockout mice by CRISPR/Cas9-mediated gene targeting. [file 43556_2022_92_MOESM2_ESM.png]

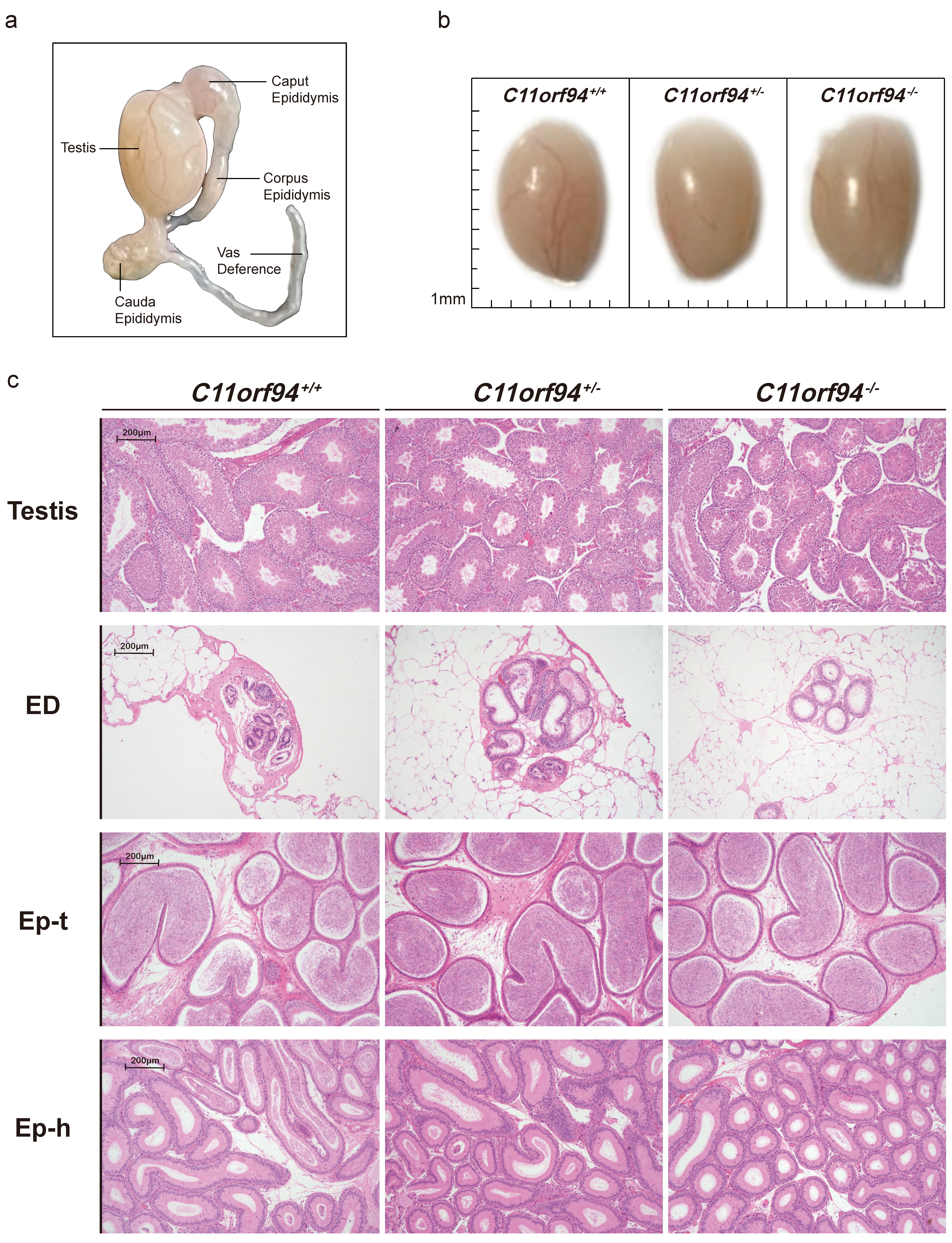

Supplement: Supplementary file 3 — Additional file 3: Figure S3. HE staining of male reproductive tissues. [file 43556_2022_92_MOESM3_ESM.png]

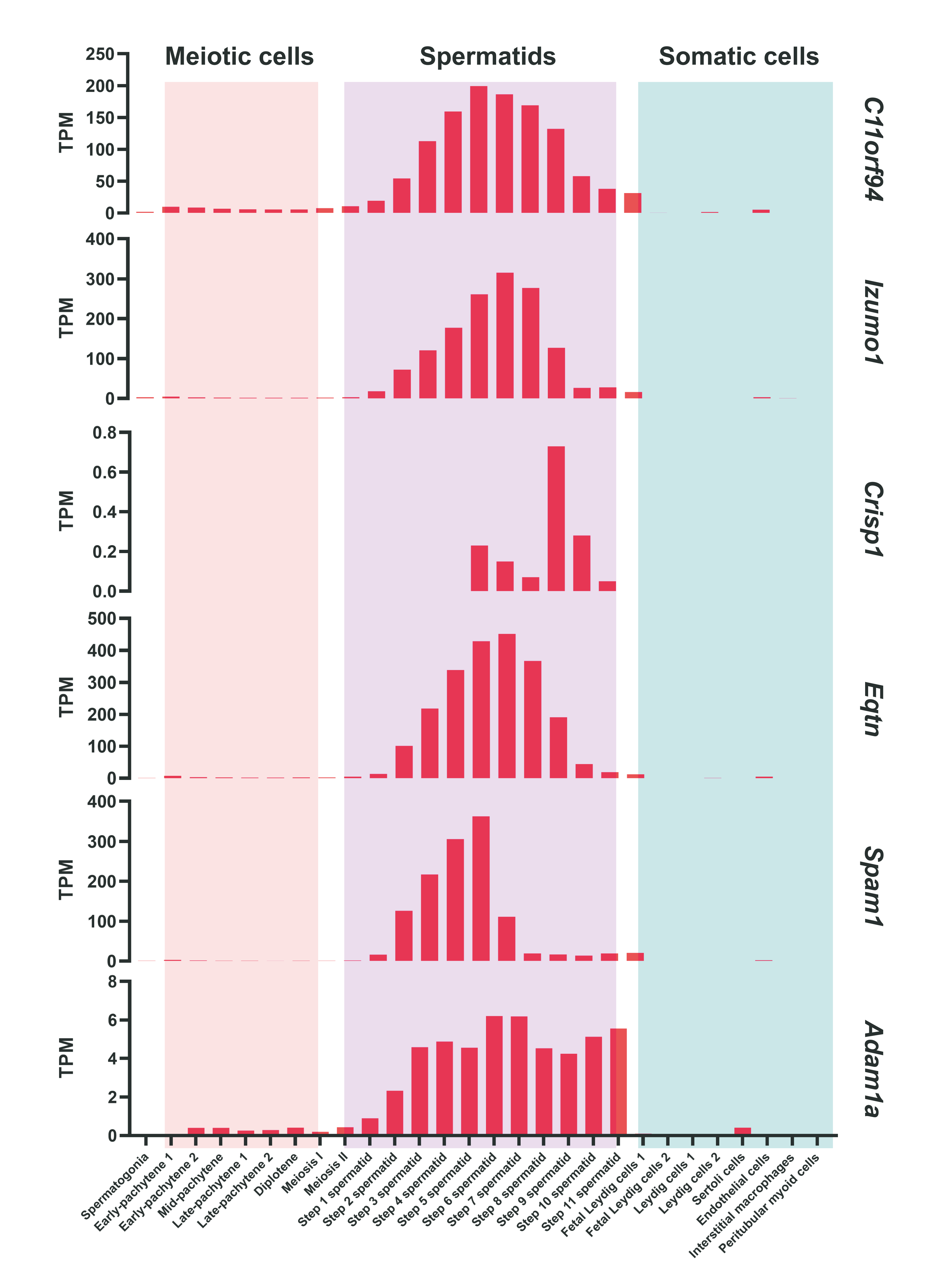

Supplement: Supplementary file 4 — Additional file 4: Figure S4. scRNA-seq data of reproductive factors’ gene. [file 43556_2022_92_MOESM4_ESM.png]
